# The Global Risk Approach Should Be Better Applied in French Hypertensive Patients: A Comparison between Simulation and Observation Studies

**DOI:** 10.1371/journal.pone.0017508

**Published:** 2011-03-03

**Authors:** Ivanny Marchant, Patrice Nony, Michel Cucherat, Jean-Pierre Boissel, S. Randall Thomas, Theodora Bejan-Angoulvant, Alexandra Laugerotte, Riad Kahoul, François Gueyffier

**Affiliations:** 1 Departamento de Pre-clínicas, Escuela de Medicina, Universidad de Valparaíso, Valparaíso, Chile; 2 IMTh – Institute for Theoretical Medicine, Lyon, France; 3 Université Lyon 1, CNRS, UMR 5558, Laboratoire de Biométrie et Biologie Evolutive, Lyon, France; 4 INSERM, CIC 201, EPICIME, Lyon, France; 5 Service de Pharmacologie Clinique, Hop L Pradel, Centre Hospitalier Universitaire Lyon, Lyon, France; 6 IR4M (UMR8081), Université Paris-Sud, CNRS, Orsay, France; 7 Novadiscovery, Lyon, France; 8 Novacare, Lyon, France; University of Modena and Reggio Emilia, Italy

## Abstract

**Background:**

The prediction of the public health impact of a preventive strategy provides valuable support for decision-making. International guidelines for hypertension management have introduced the level of absolute cardiovascular risk in the definition of the treatment target population. The public health impact of implementing such a recommendation has not been measured.

**Methodology/Principal Findings:**

We assessed the efficiency of three treatment scenarios according to historical and current versions of practice guidelines on a Realistic Virtual Population representative of the French population aged from 35 to 64 years: 1) BP≥160/95 mm Hg; 2) BP≥140/90 mm Hg and 3) BP≥140/90 mm Hg plus increased CVD risk. We compared the eligibility following the ESC guidelines with the recently observed proportion of treated amongst hypertensive individuals reported by the Etude Nationale Nutrition Santé survey. Lowering the threshold to define hypertension multiplied by 2.5 the number of eligible individuals. Applying the cardiovascular risk rule reduced this number significantly: less than 1/4 of hypertensive women under 55 years and less than 1/3 of hypertensive men below 45 years of age. This was the most efficient strategy. Compared to the simulated guidelines application, men of all ages were undertreated (between 32 and 60%), as were women over 55 years (70%). By contrast, younger women were over-treated (over 200%).

**Conclusion:**

The global CVD risk approach to decide for treatment is more efficient than the simple blood pressure level. However, lack of screening rather than guideline application seems to explain the low prescription rates among hypertensive individuals in France. Multidimensional analyses required to obtain these results are possible only through databases at the individual level: realistic virtual populations should become the gold standard for assessing the impact of public health policies at the national level.

## Introduction

### The evolving complexity of guidelines of cardiovascular prevention

The prediction of the health impact of a therapeutic strategy on a population is of growing importance to help decision-making in the prescription process and raises a number of technical issues [1]. Although cardiovascular mortality has decreased in the last decades in France, it stands second in mortality statistics [2], which explains why cardiovascular prevention remains one of the major challenges for health authorities.

Before the first recommendations for the prevention of coronary heart disease elaborated by the ESC, the European Atherosclerosis Society, and the European Society of Hypertension were published in 1994 [3], there was a plethora of confusing national and international guidelines for the prevention of CVD. The prescription of drugs was based on a univariate approach, assuming that all the benefit from treatment was explained by lowering a single risk factor. Historically, the threshold accepted to define hypertension was 160/95 mm Hg [4] until the new threshold of 140/90 mm Hg was adopted in the early 1990s by the international societies for the management of hypertension [4], [5]. As new evidence on the benefit from blood pressure lowering drugs on the incidence of cardiovascular events became available, the focus of preventive interventions changed to put emphasis on global cardiovascular risk management [3], [6], [7]. Although the estimation of total cardiovascular risk to guide patient management has been encouraged by the European guidelines since their first edition, [3] a quantitative estimate of global cardiovascular risk at the individual level was formally introduced only on 2003. Since then, the ESC practice guidelines recommend integrating the risk predicted using a multivariate model adapted to European populations [8] in the treatment decision [7]. The proposed risk equation, called SCORE, is based on data from 12 European cohorts, with 205178 individuals examined at baseline between 1970 and 1988, representing 2.7 million years of follow-up and 7934 cardiovascular deaths. The SCORE equation estimates the individual probability of experiencing cardiovascular death over 10-years resulting from combinations of covariates such as age, gender, blood pressure, total cholesterol, smoking habit and diabetic status. This change in the guideline paradigm has an intuitively obvious rationale, which is: the higher the risk, the greater the benefit from prevention, assuming the benefit to be proportional to the baseline risk. However, such a change increased drastically the complexity of the guidelines, transforming their application into a multidimensional problem.

### Are guidelines for cardiovascular prevention in hypertensives applied in France?

The paucity of information regarding hypertension management in France makes it difficult to explore the impact of recommendations on the health of the population. A few studies have revealed that the cardiovascular risk of hypertensive patients is not regularly assessed in daily practice and that when measured it does not influence physicians' practices, since the prescription of medications is mostly based on the blood pressure level of their patients [9].

### Modelling approaches as a solution to address the issue of complexity

A modelling approach has been used to quantify the treatment effect at the population level, looking at the impact of different strategies based on summarized data [10]. Working at the average level enables to reach some objectives, such as the cost effectiveness of different drug strategies in a given population. However, this approach is unable to deal with the multidimensional nature of the current guidelines, which are based both on risk and on risk factor levels.

Marshall and Rousse [11] used a hypothetical population at the general practice level to simulate and compare the impact and cost effectiveness of different screening strategies for cardiovascular prevention based on a multidimensional approach, which integrated risk factors and risk levels. In the present paper, we extend their approach, using a Realistic Virtual Population (RVP) representative of the French population [12]. We explore the impact of administering blood pressure lowering drugs to individuals of this virtual population, considered separately by gender and age categories, following specific multidimensional rules as defined in practice guidelines. Our objectives were to assess the public health impact of implementing historical and current guidelines, and to explore whether the current ones were applied in France. We provide here an estimate of the impact in terms of the number of events prevented (NEP) by treatment and of the efficiency, measured as the number of eligible subjects in the number of events prevented (NES/NEP ratio), for three different scenarios to select the individuals eligible for treatment. The results of a comparison between the theoretical implementation of current guidelines in the RVP and the observed prescriptions reported by the Etude Nationale Nutrition Santé (ENNS), suggest important discrepancies between the prescription of antihypertensive medications in France and the ESC-ESH recommendations.

## Results

### Simulated administration of antihypertensive treatment

**Figure 1:**
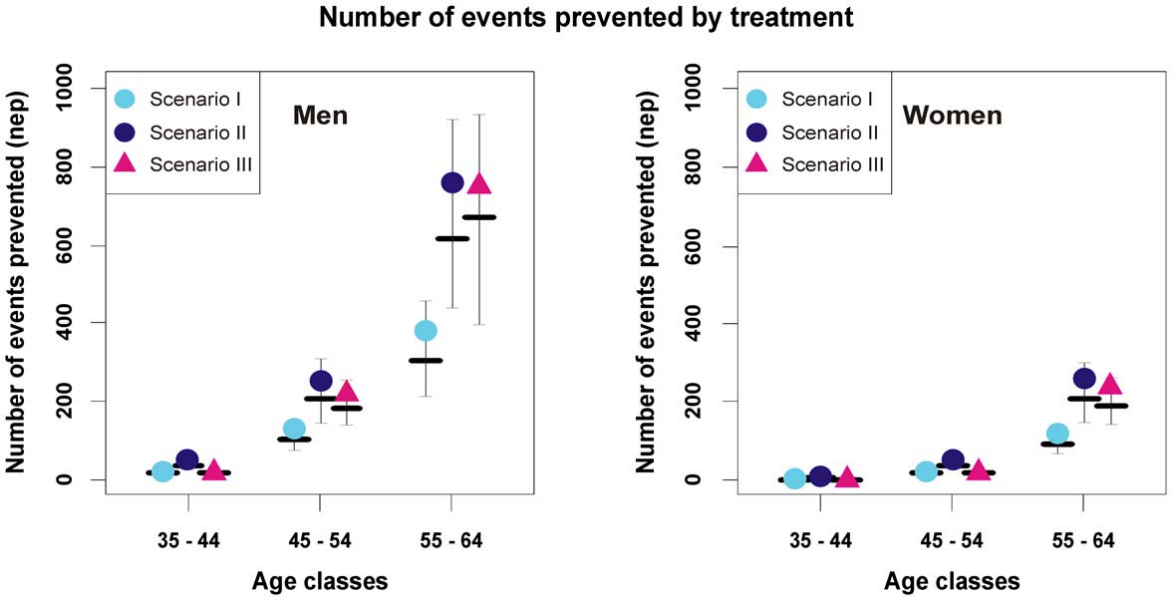
Gains from the simulation of different scenarios for treatment administration in the RVP. Points signal the number of events prevented thanks to treatment in scenarios I to III for men and women in each age category. Horizontal bars indicate the median, and vertical lines show the inter-quartile range of the predicted number of events.

The public health impact of the different scenarios in terms of NEP is presented in figure 1. Scenario II prevented twice as many accidents as scenario I; scenario III prevented a similar number of accidents as scenario II. Table 1 provides the proportion of eligible subjects in each category of age and gender and the efficiency of each scenario. Overall, the first scenario resulted in treating 12.4% of individuals; the second scenario multiplied by 2.5 the number of eligible individuals of the former, putting 31.1% of the whole population on treatment. The lower efficiency of this strategy is illustrated by the elevated NES/NEP ratio. The third scenario, integrating the absolute risk criterion, presented the smallest NES/NEP ratio. It reduced to 20.0% the number of eligible subjects, with a few women eligible for treatment under age 55 years and men under age 45 years.

**Table 1:** Proportion of eligible subjects by age and gender categories and efficiency of each scenario.

		NES/N (%)		NES/NEP
	Age classes	Men	Women	
Scenario I	35–44	10.9	3.7	
SBP≥160 or	45–54	18.0	8.5	
DBP≥95 mm Hg	55–64	22.3	14.2	
	Total	16.5	8.4	201
Scenario II	35–44	27.1	10.7	
SBP≥140 or	45–54	40.0	24.2	
DBP≥90 mm Hg	55–64	52.9	39.9	
	Total	38.7	23.7	243
Scenario III	35–44	7.2	1.6	
ESC guidelines	45–54	32.1	6.4	
implementation	55–64	49.1	35.6	
	Total	27.4	12.9	171

Abbreviations: NES, number of eligible subjects; N, size of each class;

NEP, number of events prevented.

### The theoretical guidelines implementation and the observed prescriptions

**Figure 2:**
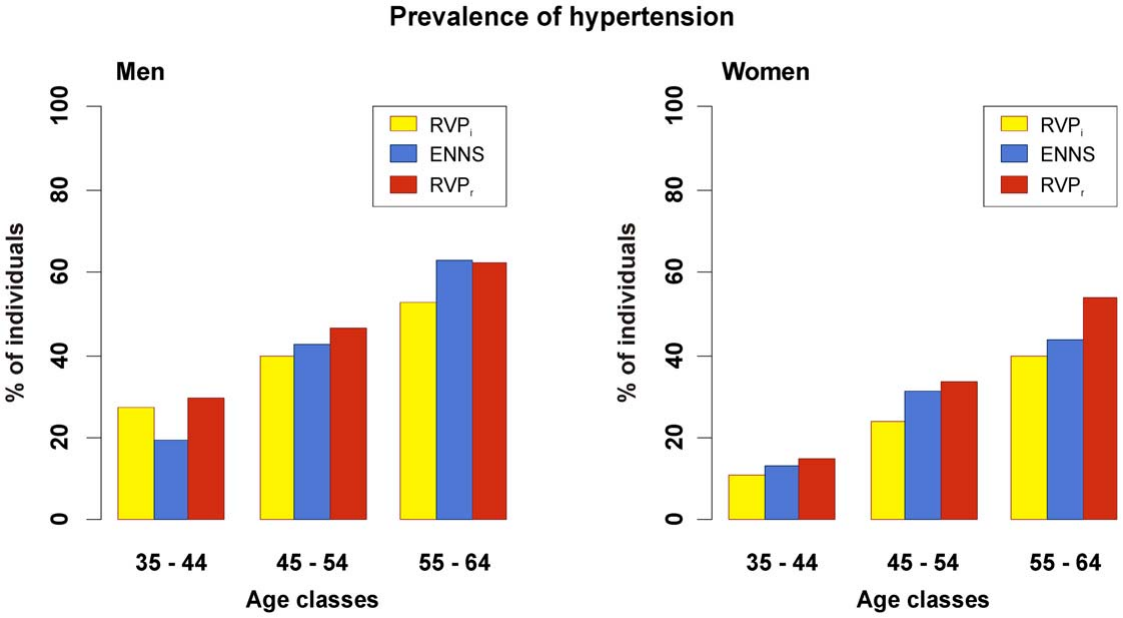
Comparison of the prevalence of hypertension estimated in the RVP with that reported by ENNS. Bars represent the percentage of hypertensive individuals in each category of age in both sexes separately, estimated in the RVP, the ENNS survey and the RVP after reconstitution of data including the proportion of hypertensive subjects taking medications in the MONICA-France cohort. Abbreviations: RVPi, the realistic virtual population initial estimates; ENNS, Etude Nationale Nutrition Santé; RVPr, the reconstituted estimates from the RVP.

**Figure 3:**
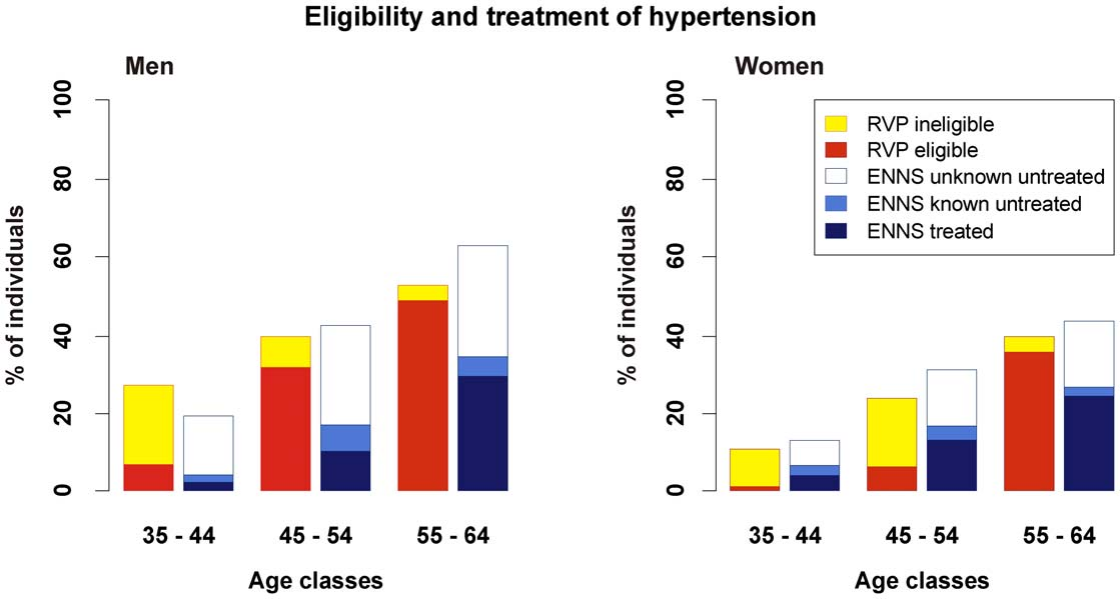
Theoretical eligibility from implementing the guidelines and treatment of hypertension in a real setting. Bars signal the proportion of hypertensive individuals eligible and ineligible for treatment according to the ESC guidelines implemented in the RVP and the proportion of hypertensive subjects that are unknown and untreated, those who are known but untreated and those who are treated in the ENNS survey for each category of age in men and women. Results are expressed as a percentage of all the individuals belonging to each class.

The prevalence of hypertension estimated in the virtual untreated individuals from the RVP was lower than that reported by ENNS in all age-sex categories except for men aged 35–44 years; and when reconstituted by integrating the information from the treated hypertensive individuals of the MONICA survey, it was higher in all categories except for men aged 55–64 years. Not taking into account treated individuals from MONICA did not significantly change the basic proportion of hypertensive individuals in the RVP, which was similar to that reported by ENNS (Figure 2). The number of treated hypertensive subjects in ENNS was low in all men and in women aged 55 to 64 years compared to the hypertensive subjects of the RVP that would be eligible for treatment according to guidelines (Figure 3). The virtual application of recommendations led to striking different proportions of eligible hypertensive individuals related to gender, resulting from the differences in absolute cardiovascular risk between men and women. No such differences were observed in ENNS.

## Discussion

Lowering the threshold to define hypertension hugely increased the potential costs of prevention, since it amplified the number of French people eligible for treatment by 2.5. Surprisingly, the impact of this “simple” shift in recommendations has never been assessed before our work. On the other hand, applying the predicted cardiovascular risk as recommended on the virtual French population significantly reduced the number of subjects eligible for treatment, especially among younger individuals because of their low risk, and was the most efficient strategy.

Our simulations illustrated how taking into account the absolute risk criterion to define the individuals eligible for treatment as in the third scenario decreases drastically the number of individuals to be treated in younger age classes (less than 1/3 of hypertensive men under age 45 years and less than 1/4 of hypertensive women under age 55 years) (Table 1, figure 3). Figure 3 also shows that hypertensive women become eligible ten years later than men, as has already been suggested by the ESC guidelines [13]. This global risk strategy, which makes treating older men preferentially because they have the highest risk, is the most efficient among the three strategies presented here, as suggested by its smallest NES/NEP ratio. Applying this strategy implies, however, that prescribers accept to exclude from cardiovascular prevention a significant proportion of the population, which is relatively protected due to age or gender. This illustration of the consequences of the high-risk strategy should thus be seen as a support for the need of complementary public health measures to reduce population levels of risk factors and delay the occurrence of cardiovascular events [13].

Despite their evident logic for optimising health resources, the ESC recommendations do not appear to be applied in France. The data on the observed prescription of antihypertensive treatments reveal that French hypertensive men of all ages and women over age 55 years are significantly undertreated with a ratio treated over eligible between 32 and 70%, whereas before 55 years of age women are overtreated with a ratio of over 200% when compared to our simulated application of guidelines (Figure 3) [14]. On the other hand, the steep increase of the eligibility proportion due to the well established risk differences related to gender and age is not reflected by ENNS, where women under 55 years are more treated than men, with a softer gradient related to age in both men and women. Of note, the largely predominant reason for not being treated is the lack of hypertension recognition, which concerns more than 65% of men under 55 years of age, leaving little room for the recommended limitation of the prescription in low risk people. These observations suggest that the absolute risk criterion is not well taken into account when deciding whether or not to use antihypertensive medications in current medical practice in France.

Our approach presents certain limitations. The age range of the RVP is restricted to 35–64 years. This was the age range in the MONICA survey, whose data constitute the core of the simulated RVP. Since the French population, as many others, is getting older, the age spectrum of the RVP should be broadened to get more comprehensive estimates of the possible consequences of implementing these scenarios in a real setting.

The RVP is composed of untreated individuals, leading to an underestimate of the prevalence of hypertension. We made this choice for the sake of simplicity, because taking into account the data of treated hypertensives in the late nineties in France [15] would imply estimating their untreated blood pressure values, introducing a source of uncertainty in the simulated population and further estimates originating from it. However, it is unlikely that differences in prevalence of this magnitude could modify significantly the proportion of eligible hypertensives resulting from the application of the CVD risk criterion, so we consider that this limitation of the RVP does not minimize the relevance of our results.

Relative risk of treatment was assumed to be constant. This may not be true and induce a bias of the predicted public health impact of the antihypertensive treatments. However, our main objective was to illustrate the different impacts of each strategy designed to identify the eligible individuals rather than to obtain an unbiased estimation of the treatment effect. The choice of a constant relative risk only simplified such a demonstration, without introducing new sources of variability that could confound the interpretation of our results.

We focused on cardiovascular death, but treatment benefit concerns also non-fatal events such as stroke, or cardiac failure, leading to a significant handicap. As non-fatal event rates depend critically upon definitions and diagnostic methods, which vary across different studies and with the development of new technologies, the SCORE project as well as the European guidelines advocates using preferably hard endpoints. In the present work, we applied the SCORE risk equations because they are more suitable for the French population than other available risk predictors, as we have shown in a previous report [12]. It would be desirable in future developments of our approach to include separate estimates for relevant outcomes, which underlines the need for high-quality data to build up suitable risk equations.

Our approach also presents original strengths. The important increase in the number of subjects eligible for treatment resulting from the change in the definition of hypertension, which has not been explored before, is directly related to the shape of the population distribution of blood pressure values: the magnitude of this change may not be the same in all other populations.

In coronary prevention, a modelling strategy has been applied to a hypothetical average patient with a known level of pre-treatment risk, who received different preventive treatments. Average estimates of costs and benefits associated with each intervention allowed to rank them according to their incremental cost effectiveness [10]. Estimates from this kind of approach are safe only when the relationship between the underlying risk factor and risk is linear, since the results computed from mean risk factors should not differ from those of averaged individual risks. However, this is not true for non-linear relationships, and the risk factor-disease incidence relation is rarely linear [16]. We have decided to work on data of each individual from the RVP, which can provide a reliable estimate of both the distribution of risk factors and cardiovascular risk. This approach makes it possible to take into account the particular risk structure of the population, which is a major advantage regarding the prediction of the public health impact of a given strategy implemented in that population. In addition, this individual data approach allows simulating complex strategies using more than one unique dimension in terms of decision threshold, as we did here in combining the level of risk, the level of blood pressure and age in the decision rule. The degree of definition of the data characterising the RVP allowed considering precise levels of each dimension defining intermediate categories of individuals. All these attributes of the RVP allow precise estimation of the number of individuals who would be concerned by a given therapeutic strategy and the potential costs of implementing that strategy.

The results of this study confirm that a modelling and simulation approach should be used in guiding public health policies, since it allows quantification of the impact of diverse rules for treatment administration before they are applied. Since the global risk approach appears to be the best scenario in terms of the benefit expected from the available health resources, the reasons why it does not influence the prescription of antihypertensive medications in France deserve further investigation.

### Perspectives

Many developments of our approach are envisaged to provide more complete estimates of the public health impact of treatment strategies. These developments are: extending the age range of the RVP, integrating the prediction of non-fatal events, regularly updating the baseline characteristics and the rate of predicted events with new information sources, taking into account compliance and treatment effect modifiers, e.g. a different relative risk in smokers. Using relevant therapeutic models could help to estimate reliably the untreated blood pressure values of treated hypertensives, allowing their integration into the RVP. Such therapeutic models could also be used to estimate precisely the treatment effect in different settings. Further developments of our approach include the exploration of alternative strategies to optimise the identification of the treatment target population, and the description of a method to design those strategies.

## Materials and Methods

**Figure 4:**
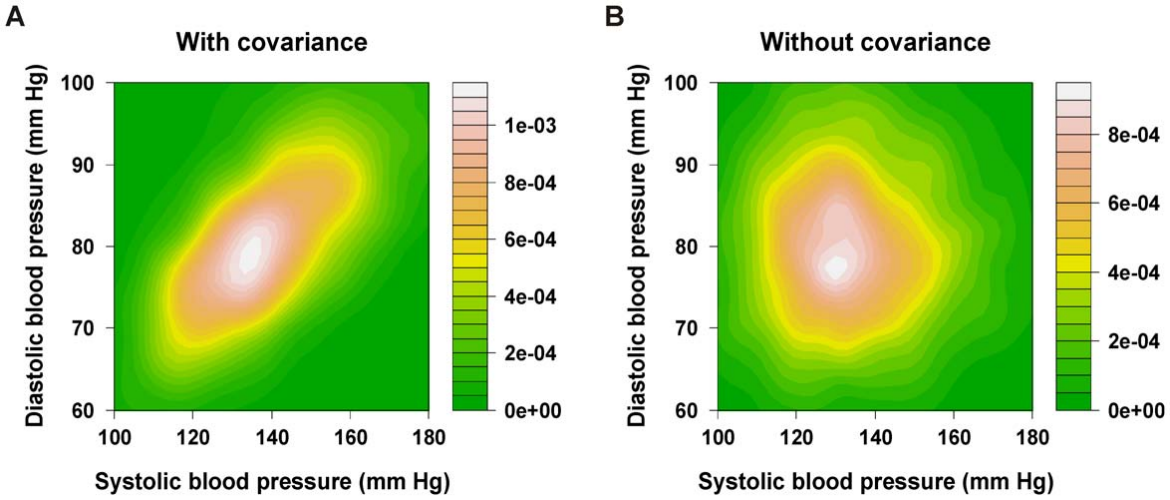
Bivariate distributions in two virtual populations, simulated with and without taking into account their covariance. Contour plots show the distribution of systolic and diastolic blood pressure on the X and Y axes, respectively. Bars beside plots indicate the scales of colours representing the density of individuals at each level of bivariate values. Panel A, taking into account the covariance. Panel B, covariance = 0.

We analysed the impact and the efficiency [17] of three strategies for administering blood pressure lowering drugs on a realistic virtual French population, following different rules of application chosen to illustrate the changes of guidelines over time. We generated the RVP [11] using the age-sex structure from official national statistics [18] to which we combined transversal data concerning the cardiovascular risk factors distribution from the MONICA-France survey [15]. This population survey of 3508 participants is the most representative study of cardiovascular risk factors in the French population. It was conducted between 1995 and 1997 in the three French registries participating in the WHO-MONICA project, including a population-based sample representative of the 35 to 64 year-old population, stratified by age and gender [19]. We used data from the untreated individuals (n = 2595) to generate the RVP through a multivariate normal distribution [20], taking into account the covariance between these characteristics. The risk distributions in different subgroups of the population with clustered risk factors are thus more realistic, and are prone to capture the important features of the risk structure of the population. This realism is illustrated through the simulated distributions of systolic and diastolic blood pressures, with and without taking into account their co-variation (Figure 4). Each individual was characterised by its age, gender, systolic and diastolic blood pressure, total cholesterol, smoking and diabetic status, from which we calculated the individual 10-year risk of fatal cardiovascular events (CVD) using the updated version of the SCORE equations for “low risk regions” [8], [21].

The size of the working sample was defined on practical bases: a sample size corresponding to 4% of the French population of the same age and sex allowed both to run programs with small computation time, and to obtain results with high enough precision.

### Simulating different scenarios for treatment administration

Virtual subjects were classified by gender and age categories of 10-year intervals. Three scenarios to select virtual subjects who would receive the simulated treatment on each category of age and sex were defined as follows:

- Scenario I, the earlier recommendation for treatment of hypertension: Subjects having a SBP≥160 or DBP≥95 mm Hg after 3 months of observation plus non-drug measures [4], [5].
- Scenario II, the current definition of hypertension: Subjects with a SBP≥140 or DBP≥90 mm Hg [4].
- Scenario III, the current ESC-ESH recommendation for CVD prevention [13], [22], applying the quantitative approach based on the predicted global cardiovascular risk and blood pressure level proposed by the ESC version of guidelines [13]. We included in this definition all those individuals having an indication such as ‘consider drugs’ or ‘drugs if persists’ in the guidelines procedure (Table 2). Thus, eligible subjects were those with stage 2 and 3 hypertension despite the level of CVD risk, stage 1 hypertension plus CVD 10-year risk 1–4%, and those with high normal blood pressure at increased risk (CVD risk ≥5%).

**Table 2:** Recommendation for use of blood pressure lowering drugs according to the blood pressure level and the level of risk.

SCORE	No hypertension		Hypertension		
10-year CVD risk	Normal	High normal	Grade 1	Grade 2	Grade 3
Low	No	No	No	Drugs	Drugs
				if persists	
Moderate	No	No	Consider	Drugs	Drugs
			drugs	if persists	
Increased	No	Consider	Drugs	Drugs	Drugs
		drugs			
Markedly	No	Consider	Drugs	Drugs	Drugs
Increased		drugs			

Schematic guidelines inspired from the table for “Management of total CVD risk – blood pressure” appeared in the ESC guidelines on CVD prevention 2007 [13].

### Size of target populations and public health impact for each scenario

The relative size of the treatment target population is given by the number of subjects eligible to treatment referred to the total number of subjects in each age-sex category. The public health impact corresponds to the number of cardiovascular deaths that would be prevented by the treatment, i.e., the NEP [23]. To express the efficiency of each strategy, we divided the number of eligible subjects by the number of events prevented (NES/NEP ratio).

Treatment effect was represented by a relative risk of 0.83 for fatal cardiovascular events for both men and women, derived from a meta-analysis of the effect of antihypertensive treatments [24]. We assumed that relative risk remains constant across time. The post-treatment risk is the initial CVD risk of each treated subject multiplied by the relative risk. The number of events corresponds to the sum of the individual risks with (NE_T_) and without (NE_baseline_) treatment. NEP was estimated by subtracting the number of events under treatment from the number of events that would have occurred in the absence of any treatment in each category of individuals.

### The virtual application of guidelines and the observed prescriptions in France

To examine whether the guidelines have influenced current medical practice, we compared the proportion of hypertensives from the RVP who were eligible to treatment according to guidelines with the observed proportion of treated individuals from the ENNS (Etude Nationale Nutrition Santé) survey [14]. This was a cross-sectional survey conducted in France in 2006–2007. In this study, blood pressure level, prevalence, awareness, treatment, and control of hypertension were described in a sample of 2266 men and women aged 18 to 74 years living in France. In order to check the reliability of the results from the RVP, we first compared the prevalence of hypertension estimated in the RVP according to its current definition with that reported by ENNS in the age range 35 to 64 years. Since the RVP was built from data of untreated individuals of the MONICA survey, we also included in this comparison the prevalence of hypertension estimated in the RVP reconstituted by taking into consideration the proportion of hypertensives receiving treatment on each class of the MONICA individuals.

**Figure 5:**
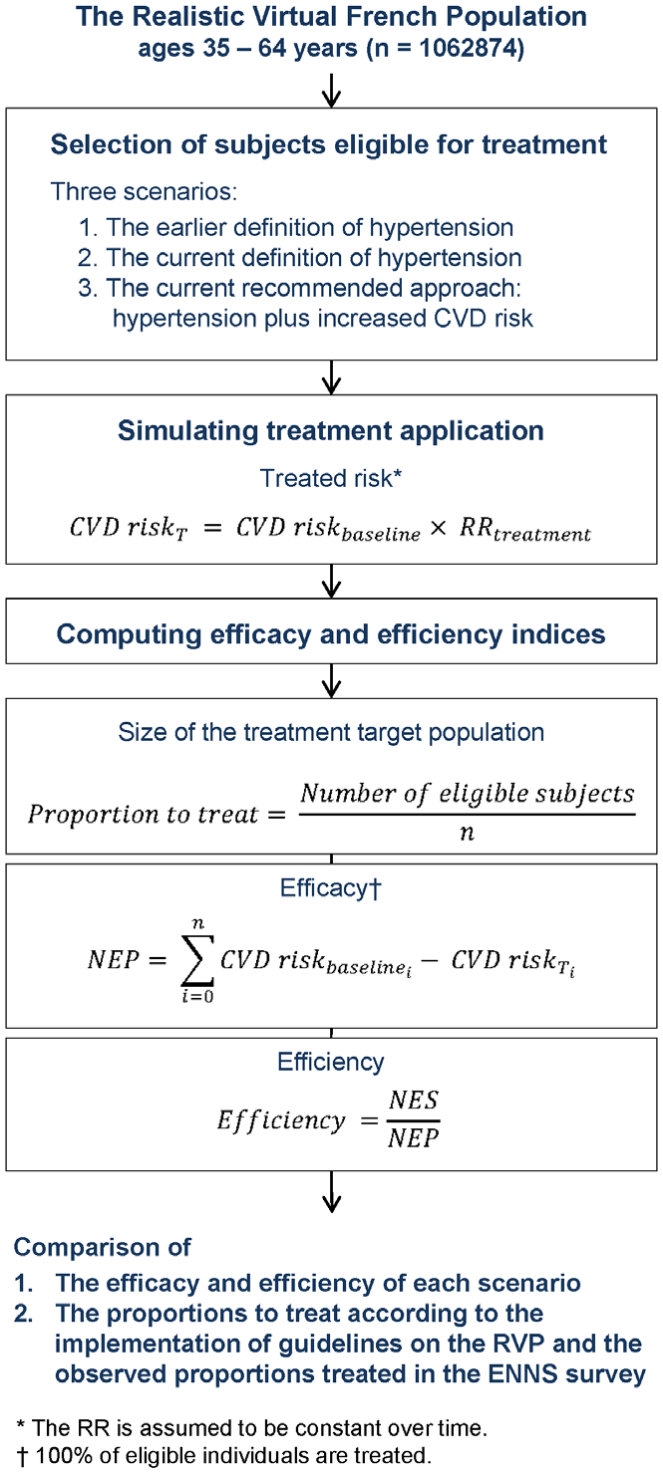
Procedure to determine the proportions eligible for treatment and the number of CVD deaths prevented in each treatment scenario. Abbreviations: CVD, cardiovascular death; NEP, number of events prevented; NES, number of eligible subjects.

An overview of the procedure used for the simulations is provided in figure 5.

All the simulations and statistical analyses were performed using R software, version 2. 7. 0 (2008) [25], [26].
